# Does implementing care farms in psychiatric hospitals prevent staff burnout? A pragmatic, mixed-method pilot study

**DOI:** 10.1186/s13104-026-07729-2

**Published:** 2026-02-23

**Authors:** Chiaki Ura, Tsuyoshi Okamura, Sachiko Yamazaki, Akira Eboshida, Yu Kawamuro

**Affiliations:** 1 Research Team for Promoting Independence and Mental Health, Tokyo Metropolitan Institute for Geriatrics and Gerontology, Sakae-cho 35-2, Itabashi-ku, Tokyo, 173-0015 Japan; 2 Bunkyo Gakuin University, Saitama, Japan; 3 Kawamuro Memorial Hospital, Niigata, Japan

**Keywords:** Burnout, Care farming, Feasibility study, Mixed methods, Psychiatric hospitals, Staff

## Abstract

**Objective:**

The purpose of this study was to implement a comprehensive intervention incorporating agricultural and horticultural activities in a psychiatric hospital, to assess the feasibility of this intervention, and to measure its impact on both staff and patients. We used a mixed-methods approach, including quantitative data from a self-administered questionnaire, qualitative data from meeting minutes, and semi-structured interviews to examine the feasibility and impact of the intervention.

**Results:**

The implementation of agricultural and horticultural activities within a hospital setting was possible by carefully listening to the preferences and opinions of the staff and by establishing step-by-step multidisciplinary meetings and projects. This approach promoted staff-led agricultural and horticultural activities. Ultimately, agricultural and horticultural activities became programs that could be implemented year-round, and collaboration with another facility was established. Over eight months, emotional exhaustion among staff also decreased, and patients provided positive feedback. The limitations of this study include its single-site design.

**Supplementary Information:**

The online version contains supplementary material available at 10.1186/s13104-026-07729-2.

## Main text

### Introduction

Care farming, which serves as a rehabilitation and social interaction avenue for individuals with dementia or mental illness, is increasingly being established as a new form of care, particularly in Europe [1]. Our previous research revealed that care farms have the potential to promote social participation and mental well-being in community-dwelling individuals with dementia and mental disorders [2, 3]. In Japan, the average age of psychiatric inpatients has been rising [4], whereas burnout [5] and poor mental health [6] among the staff at elderly care facilities have become critical issues. However, most care farm research has focused on patient outcomes, with few studies examining their effects on the mental health of staff.

The purpose of this study was to implement a comprehensive intervention incorporating agricultural and horticultural activities in a psychiatric hospital, to assess the feasibility of this intervention, and to measure its impact on both staff and patients.

## Materials and methods

The study used a mixed-methods approach, including quantitative data from a self-administered questionnaire, qualitative data from meeting minutes, and semi-structured interviews to examine the feasibility and impact of the intervention (see Table 1).

**Table 1:** Data structure used in this study

	Qualitative data	Quantitative data
A. Hospital-wide intervention	Descriptions from the meeting minutes of the project implementation committee	Self-administered questionnaires on staff members’ experiences with agricultural and horticultural activities
	Descriptions from semi-structured interviews with senior staff regarding the requirements for achieving a GCH	
B. Occupational therapy program interventions	Descriptions from semi-structured interviews with patients about their impressions of the program at the end of the intervention period	Self-administered questionnaires on staff mental well-being (WHO-5-J) and burnout symptoms(burnout symptom scale) before and after the intervention

This study was conducted at a psychiatric hospital (171 beds, 160 staff members) located in a rural district of Niigata Prefecture, Japan. This research project, launched under the slogan “Toward a Green Care Hospital (GCH),” consists of (A) hospital-wide action research using a realistic approach and (B) occupational therapy program interventions in specific wards. In (A), the following studies were conducted in consultation with researchers and hospital staff. Firstly, a project implementation committee consisting of 10 multidisciplinary staff members (nurses, occupational therapists, clinical psychologists, psychiatrists, dietitians, administrative staff) was established in September 2020, with meetings held twice a month. Secondly, a questionnaire survey was conducted with 160 staff members to determine their experiences with agricultural and horticultural activities. The original questionnaire item, ‘What agricultural and horticultural activities have you experienced so far?’, was used, with a list of major local horticultural activities provided as response options (see supplementary file for the question items). Thirdly, semi-structured interviews were conducted with five senior staff members from different wards to explore the requirements and obstacles to achieving a GCH. The questionnaire items were originally developed for this study in consultation with two researchers who had experience working in psychiatric hospitals (see supplementary file for the question items). In (B), as a pilot study, agricultural and horticultural activities were incorporated into one hour of occupational therapy once a week in a ward (with 32 patients and 28 nursing staff), where patients with relatively mild schizophrenia and depression were admitted. These activities involved cultivating rice and vegetables in the courtyard using hydroponics, followed by cooking and consumption of the harvested produce. The intervention was observed over an eight-month period. In May 2022 and February 2023, nursing staff completed a self-administered questionnaire to assess changes in mental well-being and burnout symptoms before and after the intervention. The Japanese version of the World Health Organization’s Five Well-Being Index (WHO-5-J) [7] was used to assess mental well-being, and the burnout symptom scale [8] which consists of three subscales, emotional exhaustion, depersonalization, and lack of personal accomplishment, was used to assess burnout symptoms. Patients were asked to rate the program through semi-structured interviews at the end of the intervention period in February 2023. We used the brief interview items used in our previous study [2] because patients have difficulty concentrating for long periods of time. (e.g. “Did you enjoy the program, why did you think so”) (see supplementary file for the question items).

Narratives from semi-structured interviews and the minutes of the project implementation committee meetings were subjected to descriptives analysis.

## Results

### Hospital-wide intervention

1. A questionnaire survey for staff members.

Of the 160 staff members, 119 responded to the survey (74.4% response rate), of whom approximately 70% had experience with rice or vegetable cultivation.

2) Project implementation committee meetings.

By March 2024, the project implementation committee recorded 74 meetings, guiding discussions, and decisions directed towards realizing GCH. The number of participants in the committee gradually increased to 21 staff members from all wards and departments.

3) Launching the project step-by-step.

#### Entrance project

To enable staff to experience success at an early stage, the committee first launched the “Entrance Project” to add greenery and flowers to the entrance, the face of the hospital. This resulted in positive feedback from people inside and outside the hospital (e.g., post office workers, government officials, etc.; patients said, “The entrance looks beautiful” [from the minutes of the GCH meeting]).

#### Courtyard project

Next, the hospital initiated the “Courtyard Project” to rehabilitate the neglected courtyard, previously off-limits because of overgrowth and scattered stones. Volunteers filled the courtyard with more plants and flowers, and a ramp was installed to allow safe access for staff and patients. Wheelchair users can now enjoy walking in courtyards. A grass-cutting event on September 13, 2022, involved 42 staff members who volunteered despite the event being outside their working hours.

#### Hydroponics project

In addition, a hydroponics project was set up in one ward to enable patients to enjoy indoor agricultural and horticultural activities, even during bad weather and winter. Occupational therapists, nurses, and nutritionists worked together to develop a program aimed at providing functional training in which patients harvested, cooked, and sampled vegetables that they grew. In addition, hydroponically grown basil was provided to a local employment support facility for people with disabilities and processed into basil bread. Ultimately, hydroponic projects were implemented in all three wards.

### Occupational therapy program interventions for specific wards

**Fig. 1:**
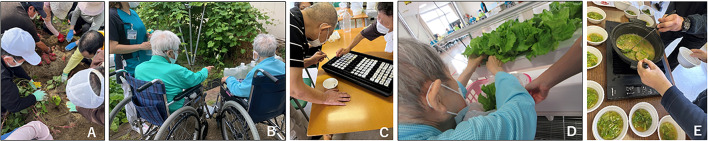
Patients’ agricultural and horticultural activities. **A** Digging sweet potatoes, **B** strolling in the courtyard in a wheelchair. **C** Making seedlings for hydrophonics. **D** Harvesting hydrophonic lettuce. **E** Cooking with harvested vegetables

In the specific ward, 10 out of 32 patients wanted to participate in the experimental activities. Ten participants consisted of 7 males and 3 females, with a mean age of 57.2 ± 11.7 years (age range from 37 to 72 years). In total, attendance at the 40 programs ranged from 29 to 40. All were long-stay patients, that is, patients who had been hospitalized for more than a year and had a schizophrenia diagnosis. However, the details were not disclosed. All seven patients who responded to the semi-structured interviews stated that they enjoyed the program and provided positive feedback. For example, statements such as “It is fun to work together with everyone (ID = 1),” “Cooking what we harvested will be useful after discharge, and I am looking forward to being discharged (ID = 2),” and “I am excited to see the growth of the rice and vegetables (ID = 3)” were observed. Among the nursing staff (24 valid responses; seven males; 62.5% in their 50s and 60s), the repeated-measures t-test revealed a significant decrease in emotional exhaustion, a subscale of burnout symptoms (t (20) = 2.387, *p* = 0.027). There were no significant differences in the scores of the other burnout subscales and the WHO-5-J (Fig. 1).

## Discussion

In this study, the implementation of care farming activities in a hospital setting was made possible by carefully listening to the preferences and opinions of the staff and establishing multidisciplinary meetings and projects. This approach boosts staff morale and promotes staff-led agricultural and horticultural activities. The process suggested that achieving a full-scale GCH was not feasible in the short term; instead, beginning with manageable activities while fostering better communication among staff is essential. For example, the grass-cutting activity presented potential for team building, and initiating the project with greening efforts at the hospital entrance, a central area, provided a sense of accomplishment. Ultimately, agricultural and horticultural activities became programs that could be implemented year-round, and collaboration with another facility was established. Emotional exhaustion among staff members has also decreased. Previous research suggests that care farms may help alleviate depression and anxiety [1], indicating that care farming activities could serve as innovations to address stress and turnover among caregiving staff.

### Limitations

This study has several limitations, including its implementation at a single hospital and a small sample size. Future research should incorporate controlled intervention studies to further validate the findings. Additionally, this study was not based on a specific hypothesis, but was driven by practical clinical motives to improve the environment for patients and assess feasible outcomes while gaining staff support—an approach rarely seen in Japanese medical settings. Nonetheless, it provides practical insights for implementation in similar contexts. Future studies should be grounded in clearly defined research questions and hypotheses.

## Supplementary Information

Below is the link to the electronic supplementary material.

Supplementary Material 1

## Data Availability

The datasets used and/or analyzed in the current study are available from the corresponding author upon reasonable request.
