# Supplementary material for: Does implementing care farms in psychiatric hospitals prevent staff burnout? A pragmatic, mixed-method pilot study
Source: BMC Res Notes. 2026 Feb 23;19:148. doi: 10.1186/s13104-026-07729-2 (PMC13037029; doi:10.1186/s13104-026-07729-2)
Supplement: Supplementary file 1 — Supplementary Material 1 [file 13104_2026_7729_MOESM1_ESM.docx]

The list of question items

1. The question item of the questionnaire survey conducted with 160 staff members (Line 71)

‘What agricultural and horticultural activities have you experienced so far?’

Answer options

1. Cultivation of rice
2. Vegetable/fruit cultivation
3. Growing flowers/foliage plants
4. Flower arranging/decorating
5. Other
6. None of the above
7. The question item of the semi-structured interviews conducted with five senior staff members (Line 74)
8. ‘What do you need to achieve a GCH in this hospital?’
9. ‘What do you think are the obstacles to achieve a GCH in this hospital?’
10. The question item of the semi-structured interviews conducted with patients after the intervention for 8 month (Line 88)
11. ‘Did you enjoy the program, and why did you think so?
12. ‘Did the program change your live?’
